# Supercritical methane adsorption measurement on shale using the isotherm modelling aspect

**DOI:** 10.1039/d2ra03367d

**Published:** 2022-07-15

**Authors:** Aminah Qayyimah Mohd Aji, Dzeti Farhah Mohshim, Belladonna Maulianda, Khaled Abdalla Elraeis

**Affiliations:** Universiti Teknologi PETRONAS 32610 Seri Iskandar Perak Malaysia aqayyimah@gmail.com +60-13-7337671; Universiti Teknologi Malaysia Skudai Johor Malaysia; PT. Pertamina Jl. Medan Merdeka Timur 1A Jakarta 10110 Indonesia

## Abstract

In shale gas reservoirs, adsorbed gas accounts for 85% of the total shale gas in place (GIP). The adsorption isotherms of shale samples are significant for understanding the mechanisms of shale gas storage, primarily for assessing the GIP and developing an accurate gas flow behaviour. Isothermal adsorption experiments primarily determine the adsorption capacity of methane in shale gas reservoirs. However, experimental data is limited due to the heterogeneous properties of shale and extreme reservoir conditions at high pressures and temperatures. This work discusses the effect of total carbon (TOC), pore size distributions, and mineralogical properties on adsorption capacity. In this study, the gravimetric adsorption isotherm measurement method was applied to obtain the adsorption isotherms of methane on four shale core samples from Eagle Ford reservoirs. Four shale core samples with TOC of 9.67% to 14.4% were used. Adsorption experiments were conducted at a temperature of 120 °C and to a maximum pressure of 10 MPa. The data obtained experimentally were compared with adsorption isotherm models to assess each model's applicability in describing the shale adsorption behaviour. A comparison of these models was performed using fitting and error analysis. It was observed that the calculated absolute adsorption of supercritical methane is higher than the excess adsorption. The percentage of differences between the absolute and excess adsorption is more significant at a pressure higher than the critical methane pressure of 9.6%. Sample EF C has the highest adsorption capacity of 1.308 mg g^−1^, followed by EF D 1.194 mg g^−1^, EF B 0.546 mg g^−1^, and EF A 0.455 mg g^−1^. Three statistical error analyses, average relative error (ARE), the Pearson chi-square (*χ*^2^) test and root mean square error (RMSE) deviation were used to assess the applicability of each model in describing the adsorption behaviour of shale samples. The order of adsorption isotherm fitting with experimental data is Toth > D–R = Freundlich > Langmuir. Error analysis shows that the Toth model has the lowest values compared to other models, 0.6% for EF B, 2.5% for EF C, and 2.2% for EF A and EF D, respectively.

## Abstract 2

Adsorption isotherm can be used to depict the adsorption in shale.

## Graphical abstract

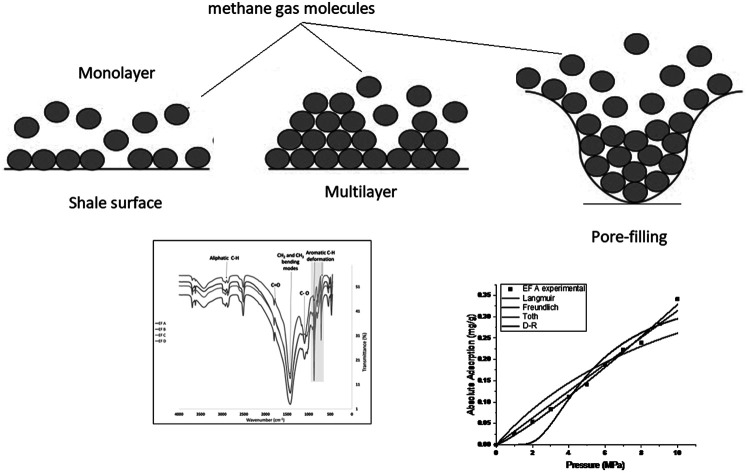

## 1 Introduction

In the last few decades, the global energy landscape has shifted dramatically. The expanding economies are responsible for the rapidly increasing need for energy sources. As a result, energy consumption rises in tandem with economic expansion and the availability of new energy sources.

In 2016, fossil fuels made up 85% of world energy demand, with oil (33%), coal (28%), and natural gas (24%) making up the top three rankings.^1^ However, in recent years, natural gas production has grown remarkably, with over 2.6% average annual growth rate over the past ten years compared to other fossil fuels such as oil at 1.3% and coal at 0.8%.^2^ The surge in natural gas production was driven by unconventional gas resources such as shale gas.

Shale gas is the fastest-growing natural gas resource in the United States and worldwide. According to the Energy Information Administration (EIA), in 2019, the estimates of proved reserves in the United States account for 495.4 tcf.^3^ The rapid increase in natural gas production from shale formations resulted from advanced extraction technology such as hydraulic fracturing and horizontal drilling.^4^

Shale gas is the natural gas trapped within shale formations.^5^ Shales are fine-grained (<62.5 μm) sedimentary source rocks with complex mineralogy consisting of organic and inorganic matters.^6^ Consequently, shale reservoir is heterogeneous and influenced by the shale environment's climate, deposition, and diagenesis.^7,8^

Unlike conventional reservoirs, shale has low porosity (less than 10%) and low permeability (nanoDarcy) due to the complex microstructures and pore systems.^9–12^ As a result, porous shale media predominantly consists of interparticle (between mineral particles), intraparticle (within mineral particles), and organic pores, with diameters ranging from nanometre to micrometre.^13^

The natural gas in shale is stored in three ways: free gas; within the rock pores and natural fracture, as adsorbed gas; primarily on organic materials and clay, and as dissolved gas; in the organic materials.^14–16^ In shale gas reservoirs, the adsorbed gas made up 20–85% of total gas capacity, with methane as the significant gas composition.^10^

Gas adsorption in organic-rich shale is a comprehensive process controlled by various factors. Due to the complex and heterogeneous characteristics owned by the shale matrix, quantifying the weight of each element is challenging. Nevertheless, understanding shale adsorption behaviour is critical for determining the gas in place (GIP) and predicting accurate gas flow behaviour, particularly during production.

### 1.1 Adsorption in shale

Adsorption in shale formation occurs through the physical adsorption process or “physisorption”.^17^ The gas molecules adhere to the shale surface by the van der Waals forces. Hence, the adsorption strongly depends on methane and shale surface properties along with pressure and temperature.^18^ Adsorption in shale is controlled by the geochemical characteristics, mineralogical composition, pore structures, and reservoir conditions (pressure and temperature).^19–22^ In shale gas reservoirs, methane adsorption generally occurs at high temperature and pressure, higher than critical methane temperature at 190.55 K and pressure at 4.59 MPa, indicating that methane in shale behaves as supercritical fluid.^23^

The adsorption phenomenon of methane on shale surfaces has been described experimentally, including monomeric, volumetric, and gravimetric methods. These methods constructed the adsorption isotherms and evaluated methane adsorption capacity.^23^ Numerous experimental results for methane and shale adsorption isotherms have been reported, describing the properties governing the adsorption capacity in shale.^16,21,24–31^ The shale organic matter is associated with the *in situ* generation of hydrocarbons within shale.^32^ Commonly organic matter in shale is denoted by the total organic carbon (TOC) content. Large amounts of methane are adsorbed on nanopores within organic matter in shale. The presence of organic matter in shale increases porosity and imparts anisotropy, thus facilitating adsorption.^16^

A positive correlation between the TOC and methane adsorption capacity of shale has been observed in previous studies that have been conducted.^16,25,33,34^ In their work, Ross and Bustin (2009) indicated that methane adsorption in Devonian shale linearly increases with the TOC, citing that the organic fraction is a primary control of the adsorption.^34^ In other work, the micropore volume of Lower Silurian shale increased linearly with TOC content, depicting TOC as the critical factor in controlling the micropore structure.^35^ These micropores are vital for porous media, providing high surface areas and greater adsorption energies.^16^

Other than organic matter, shale also contains clay minerals. Jin and Firoozabadi (2014) observed that adsorption also occurs on clay surfaces.^36^ The adsorption of methane is related to clay interactions, and the cation exchange in clay is the main contribution toward adsorption. However, further findings indicate that the hydrophilic nature of clay minerals absorbs the water molecule filling pore throats, thus reducing the available adsorption site for methane molecules.^37,38^ Li *et al.* (2015) conducted a study based on a parallel experiment to determine the key factors governing the adsorption capacity of shale, exhibiting a stronger positive linear correlation with TOC compared to other parameters.^26^

However, most of the experiments conducted did not replicate shale conditions. Shale formations' depths are more than 3000 m, with an average reservoir temperature of more than 100 °C and pressure higher than 27 MPa.^22,39^ Furthermore, due to the limitations of methane adsorption instruments, there is limited data for the study, challenging an accurate methane adsorption mechanism for shale. Some researchers used a molecular simulation approach to simulate methane behaviour in shale. However, most simulations were simplified, and homogenous pore structures do not represent the heterogenous properties of shale.^22^ Due to the complex characteristics of shale, the adsorption isotherms measurements were always coupled with an empirical equation called the adsorption isotherm model for accurately describing the adsorption behaviour of methane in shale.

### 1.2 Adsorption isotherm model

The adsorption isotherm is a measurement that determines the adsorption capacity of gas or liquid on a solid surface at varying pressure and a constant temperature. Adsorption isotherm provides information on the maximum amount of gas adsorbed on porous shale surfaces.^40^ The adsorption isotherm model generated based on the gas adsorption on the solid surface based on several theoretical assumptions has been used to depict the adsorption behaviour of methane with shale.^23,41–44^ The adsorption isotherm model is an essential part of the studies and is used in shale gas reservoir simulators to forecast reservoir production.^42,45^

Adsorption isotherm models can mainly be divided into monolayer adsorption models (Langmuir and Freundlich isotherm models), multilayer adsorption models (Toth isotherm model), and pore-filing models (Dubinin–Radushkevich (D–R) isotherm model)^46^ The description of each model is further explained below.

#### Langmuir isotherm model

The Langmuir isotherm is the most prominent and widely applied equation to describe adsorption equilibrium. Langmuir isotherm explains the increasing surface occupancy as a function of pressure until the entire surface area is coated with a single layer of molecules and no further adsorption can occur.^47^ Langmuir's model can be shown in the following form eqn (1),^48^1
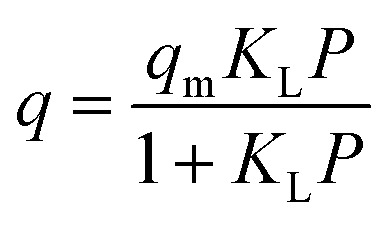
where *q* is the adsorbed amount under equilibrium temperature and pressure, *q*_m_ is the maximum adsorbed capacity, *P* is the adsorption pressure (MPa), *K*_L_ is the Langmuir constant (L mg^−1^).

Langmuir isotherm model has been used regularly to model methane adsorption in coal and shale reservoirs.^49–53^ Commercial reservoir simulators employed the Langmuir isotherm to generate the shale gas phase behaviour and gas production models.^54–58^ However, this model has shown limitations in predicting the adsorption isotherm of methane with shale, especially at high pressure and temperature, which resulted from the theoretical assumptions of homogenous and isothermal assumptions.^59,60^ Therefore, evaluating the performance of different adsorption models for methane adsorption in shale is essential.

#### Freundlich isotherm model

The Freundlich model is an empirical equation describing gas phase adsorption on the surface of the solid adsorbent and pressure.^61^ Freundlich isotherm model has been employed to characterise the heterogeneity of the adsorbent surface.^23,44,62,63^ The equation is described in eqn (2).2
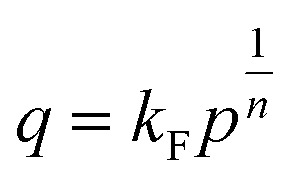
where *k*_F_ is defined as the Freundlich isotherm constants related to adsorption capacity (mg g^−1^), *n* is the adsorption intensity, and *P* is the adsorption pressure (MPa).

#### Toth isotherm model

Toth isotherm model is another empirical model equation that uses the power function of the relation between adsorption capacity and the adsorption potential of the adsorbent surface.^64^ This model derives from the adsorption potential theory, generally used to describe heterogeneous adsorption.^48^ Toth isotherm model is described in eqn (3).3
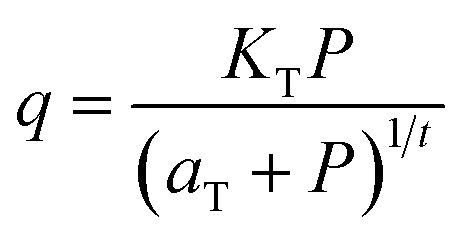
where *q* is the adsorbed amount (mg g^−1^), meanwhile *k*_T_, *a*_T_*t* is defined as the Toth isotherm constants, and *P* is the adsorption pressure (MPa).

#### Dubinin–Radushkevich (D–R) isotherm model

This model is derived from Polanyi adsorption potential theory, based on the assumption that the adsorption process was related to micropore volume filing instead of molecular-layer adsorption on pore walls.^65^ Literature has shown that the D–R model has been used to estimate shale gas's high-pressure methane adsorption capacity and has demonstrated its consistency with the calculations based on micropore volumes.^66–69^ The D–R equation is expressed as follows eqn (4);^70^

4 *q* = *q*_s_ exp{−*k*_ad_*ε*^2^}

where *q* is the adsorbed amount under equilibrium temperature and pressure, *q*_s_ is the theoretical isotherm saturation capacity (mg g^−1^), *k*_ad_ is D–R isotherm constant (mol^2^ kJ^−2^), and *ε* is D–R isotherm constant. The parameter *ε* can be correlated as eqn (5);5
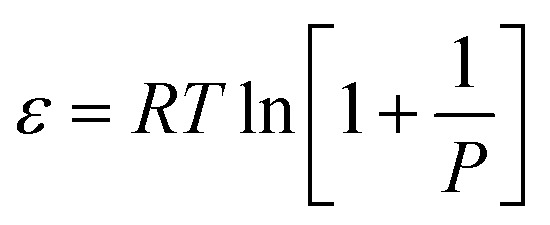
where *P* is the pressure (MPa), *R* is the ideal gas constant (0.008314 kJ (mol^−1^ K^−1^)), and *T* is the temperature (K).

### 1.3 Motivation for the study

High-pressure methane adsorption studies have found a positive correlation between the adsorption capacity and shale's total organic content (TOC). Organic matters hold a huge volume of micropores with a relatively large surface area, thus facilitating gas adsorption. Many studies have demonstrated that the TOC of gas-bearing shales in the United States varies significantly among different bed layers and basins, from 1.5% to 25%.^71^ The TOC content varies widely among shale reservoirs and within a formation itself.

However, to date, discussion on the effect of TOC on the adsorption capacity of shale is relatively inadequate. As a result, the research on methane adsorption at high temperatures is minimal, becoming one of the driving forces behind this study. Furthermore, research into proper adsorption isotherm model fitting methane at different TOC levels is currently lacking.

This paper compares empirical isotherm models with experimental gravimetric measurements on four Eagle Ford shale samples with different TOC. Four adsorption isotherm models are compared statistically to present the specific characteristics of each model. This comparison shows the best adsorption isotherm models, especially for shale gas engineering uses.

## 2 Adsorption isotherm measurements

### 2.1 Materials

Four shale samples were retrieved from the Eagle Ford shale formations in Texas, United States, with an average reservoir temperature of 120 °C. Methane gas with purities of 99% was used to avoid any uncertainty in the measurements due to the gas impurity.

### 2.2 Shale core samples characterisation

Shale samples were characterised to determine their total organic carbon (TOC) content, chemical compositions, mineralogical compositions, pore size distribution, and specific surface area (SSA). The TOC measurements were conducted using the TOC analyser. The instruments determined the organic and inorganic carbon content through the amount of CO_2_ released during the combustion.^72^

The mineralogical constituents of the shale core samples were characterised using the X-ray diffraction (XRD) instrument. The measurements were conducted with scattering angles 2*θ*, between 5° and 75° with a step size of 0.05° s^−1^ using CuKα radiation (*λ* = 1.54 Å).^73^

The low-pressure nitrogen (N_2_) adsorption–desorption was used to obtain the shale pore systems at 77 K (−196 °C). The core samples specific surface area and pore size distribution were calculated using Braunauer–Emmet–Teller (BET) and Barrett–Joyner–Halenda (BJH).^74,75^

Elemental analysis of organic matter was performed using a Shimadzu FTIR microscope in the ATR and KBR mode. The spot size for all measurements was 100 × 100 μm. The spectral window ranged from 700 to 4000 cm^−1^, with a resolution of 4 cm^−1^. FTIR spectra were baseline-corrected, the absorption band intensity was normalised before use in model development or property prediction, and no pre-treatment was performed prior to measurement. Infrared spectra were used to estimate the amount of organic matter by looking at the C–H and C–C stretches of aliphatic and aromatic carbon.

### 2.3 Gravimetric adsorption isotherms measurement

The high-pressure adsorption isotherm experiments were conducted by using the gravimetric adsorption instrument. The adsorption test for all samples was carried out at maximum pressure of 10 MPa and a temperature of 120 °C. The excess adsorption amount was obtained following eqn (6).6
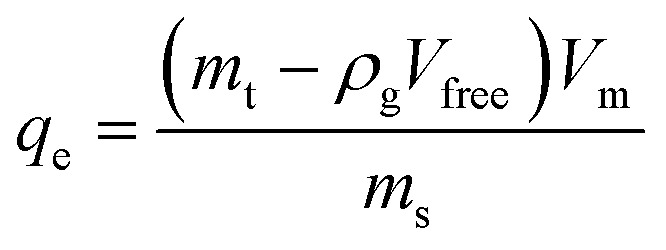
where *q*_e_ (mg g^−1^) is the excess adsorption capacity, *m*_t_ is the total mass of methane adsorbed (mg), *m*_s_ mass of the shale samples (mg), *ρ*_g_ is the bulk density of methane, *V*_free_ is the void volume, and *V*_m_ is the methane molar volume at the standard condition.

Experimentally, the adsorption quantity measured was the excess adsorption quantity called Gibbs's excess adsorption.^30^ Thus, it is necessary to convert the excess values to absolute adsorption (*q*_m_) before fitting the experimental value with the adsorption isotherm. Therefore, the following equation was used to get the *q*_m_ based on the experimental measurements. Absolute adsorption isotherms were expressed in the functions of Gibbs excess adsorption isotherms as follows (eqn (7)):7
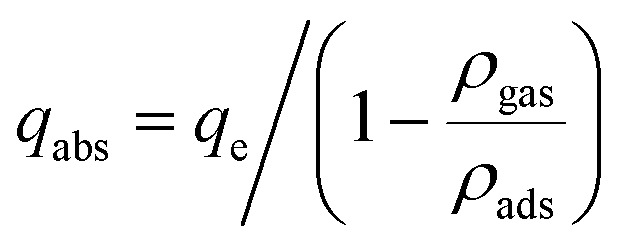
where *q*_e_ is the surface excess adsorption amount, *q*_abs_ is the absolute adsorption amount (mg g^−1^), *ρ*_gas_ is the density of free gas (g cm^−3^), and *ρ*_ads_ is the density of adsorbed gas (g cm^−3^). The *ρ*_gas_ is calculated from the National Institute of Standards and Technology (NIST). Foremost, the value of the adsorbed gas density (*ρ*_gas_) is required to calculate the value of absolute adsorption. In this work, the liquid methane density is considered as the *ρ*_ads_ taken as 0.421 g cm^−3^.

## 3 Model evaluation

The data obtained experimentally were compared with adsorption isotherm models to assess each model's applicability in describing the shale adsorption behaviour. In addition, non-linear regression and error analyses were carried out to compare various adsorption isotherm models and identify the best fit for the experimental data. Both methods have been the most feasible tools for expressing the best-fitting relationship, mathematically analysing the adsorption systems, and validating an isotherm model's consistency and theoretical assumptions.^70^

In this work, the experimental data were fitted non-linearly with different adsorption models, and the model parameters were obtained. The Pearson chi-square (*χ*^2^) test and root mean square error (RMSE) deviation are two well-known statistics for evaluating a regression model's goodness of non-linear fit. These statistics can determine whether the observed data originated from an experiment in which the fitted model is accurate.

The *χ*^2^ test statistic is the sum of the squares of the differences between the experimental data obtained from models, with each squared difference divided by the corresponding data obtained by calculating from models, the mathematical expression of *χ*^2^ shown in eqn (8).8
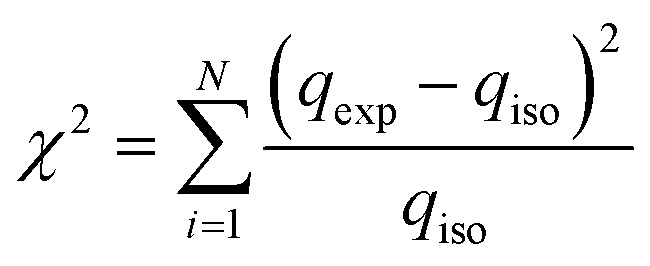
*q*_exp_ and *q*_iso_ are the equilibrium capacity (mg g^−1^) obtained from the experiment and isotherm model. A small *χ*^2^ value indicates its similarities, while a more significant number represents the variation of the experimental data.

The RMSE is the standard deviation of the prediction values with the observed value. The residuals measure how dispersed these residuals are to the data points on the regression line.^70,76^ The RMSE is expressed as eqn (9).9
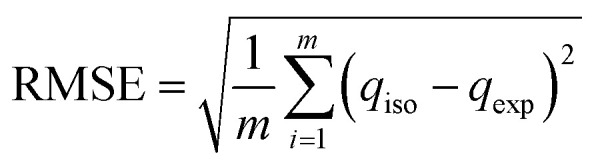


In this work, we considered the average relative error (ARE) to evaluate the disagreement between the model fitted of absolute adsorption with the experimental results (eqn (10)).10
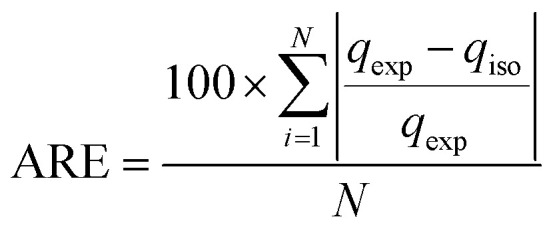
where *q*_exp_ is adsorption value obtained through experiments measurement, while *q*_iso_ is the value calculated through adsorption models.

## 4 Discussion

### 4.1 Shale core samples characterisation

**Fig. 1:**
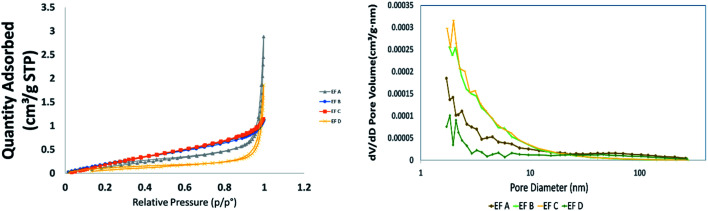
(a) The low-pressure nitrogen adsorption–desorption isotherms for the shale samples, (b) BJH pore size distributions for Eagle Ford shale samples.

**Fig. 2:**
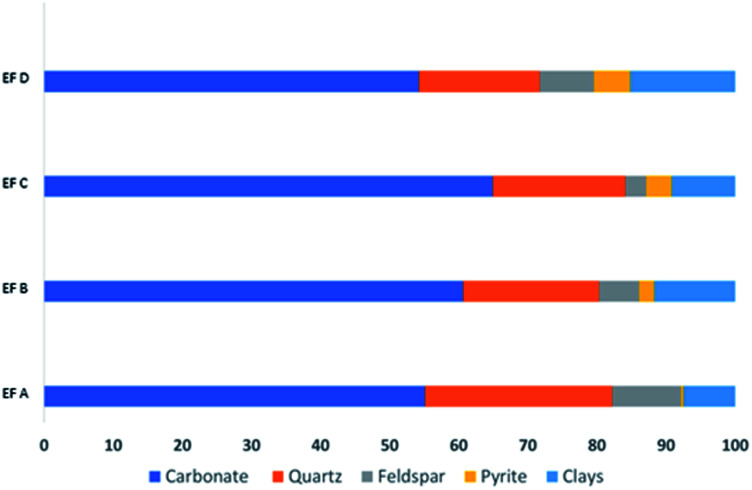
Mineral compositions of Eagle Ford shale samples.

The results of shale sample characterisations are listed in Table 1. The TOC values obtained for sample EF C are 14.4%, sample EF D is 10.7%, sample EF A is 10.4%, and sample EF B is 9.69%. The pore size distribution and SSA were obtained using the BET and BJH equation from the low-pressure nitrogen adsorption measurement. Fig. 1 shows the graphs obtained from the measurement. The pore size distribution curves display the unimodal distribution, with the peak at approximately 2–10 nm for all samples. As illustrated in Fig. 1(b), it was revealed that pores with widths between 2 and 20 nm mainly contribute to the total pore volumes. According to the International Union of Pure and Applied Chemistry (IUPAC) classification, pore sizes with widths between 2 nm and 20 nm are classified as mesopores.^77^ This result is consistent with a measurement conducted by Ojha *et al.* (2017) on Eagle Ford shale (Fig. 2).^78^

**Table 1:** TOC, SSA, and pore size distribution in Eagle Ford shale samples

Samples	TOC (%)	BET surface area (m^2^ g^−1^)	BJH adsorption cumulative pore volume (cm^3^ g^−1^)	Average pore width (nm)
EF A	10.4	0.867	0.003345	16.305
EF B	9.7	0.833	0.001487	5.766
EF C	14.4	1.042	0.001567	6.019
EF D	10.7	0.936	0.002142	18.305

**Fig. 3:**
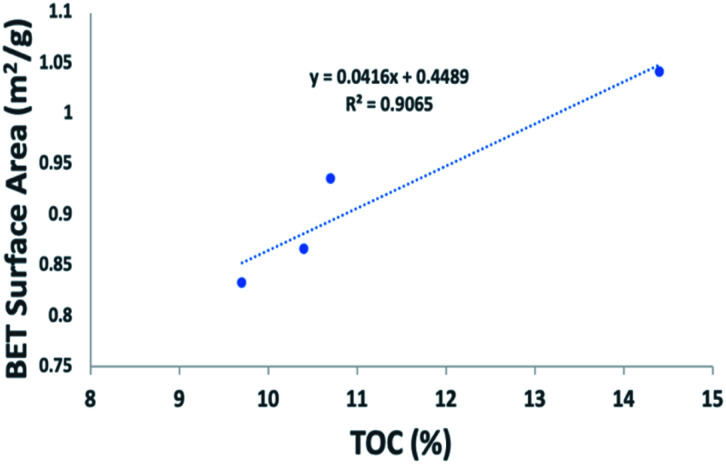
Correlations between the TOC and SSA (m^2^ g^−1^).

The SSA of each sample shows that EF C has the largest surface area with 1.1042 m^2^ g^−1^, followed by EF D (0.9360 m^2^ g^−1^), EF A (0.8674 m^2^ g^−1^), and EF B with 0.8326 m^2^ g^−1^. The shale samples have abundant nanopores, and most pores are in organic matter. Organic matter provides a huge SSA, creating a favourable condition for adsorption. The SSA also positively correlates with TOC (Fig. 3), creating favourable gas adsorption and storage conditions.

The XRD results show that calcite is the dominant mineral in all samples. The mineral distributions in all the samples are shown in Fig. 3. For example, sample EF C has the highest calcite minerals with 63.6%, quartz with 19.2%, feldspar and clay with 3% and 9.2%. These findings followed the previous results on Eagle Ford mineral deposition. The Eagle Ford is a carbonate-rich shale hydrocarbon play where mineralogy based on XRD consists of; 40–60% calcite, 10–30% quartz, and clays with less than 20%.^79^

The XRD results show that calcite is the dominant mineral in all samples. The mineral distributions in all the samples are shown in Fig. 3. For example, sample EF C has the highest calcite minerals with 63.6%, quartz with 19.2%, feldspar and clay with 3% and 9.2%. These findings followed the previous results on Eagle Ford mineral deposition. The Eagle Ford is a carbonate-rich shale hydrocarbon play where mineralogy based on XRD consists of; 40–60% calcite, 10–30% quartz, and clays with less than 20%.^79^

### 4.2 Adsorption isotherms measurements

#### Excess and absolute adsorption

**Fig. 4:**
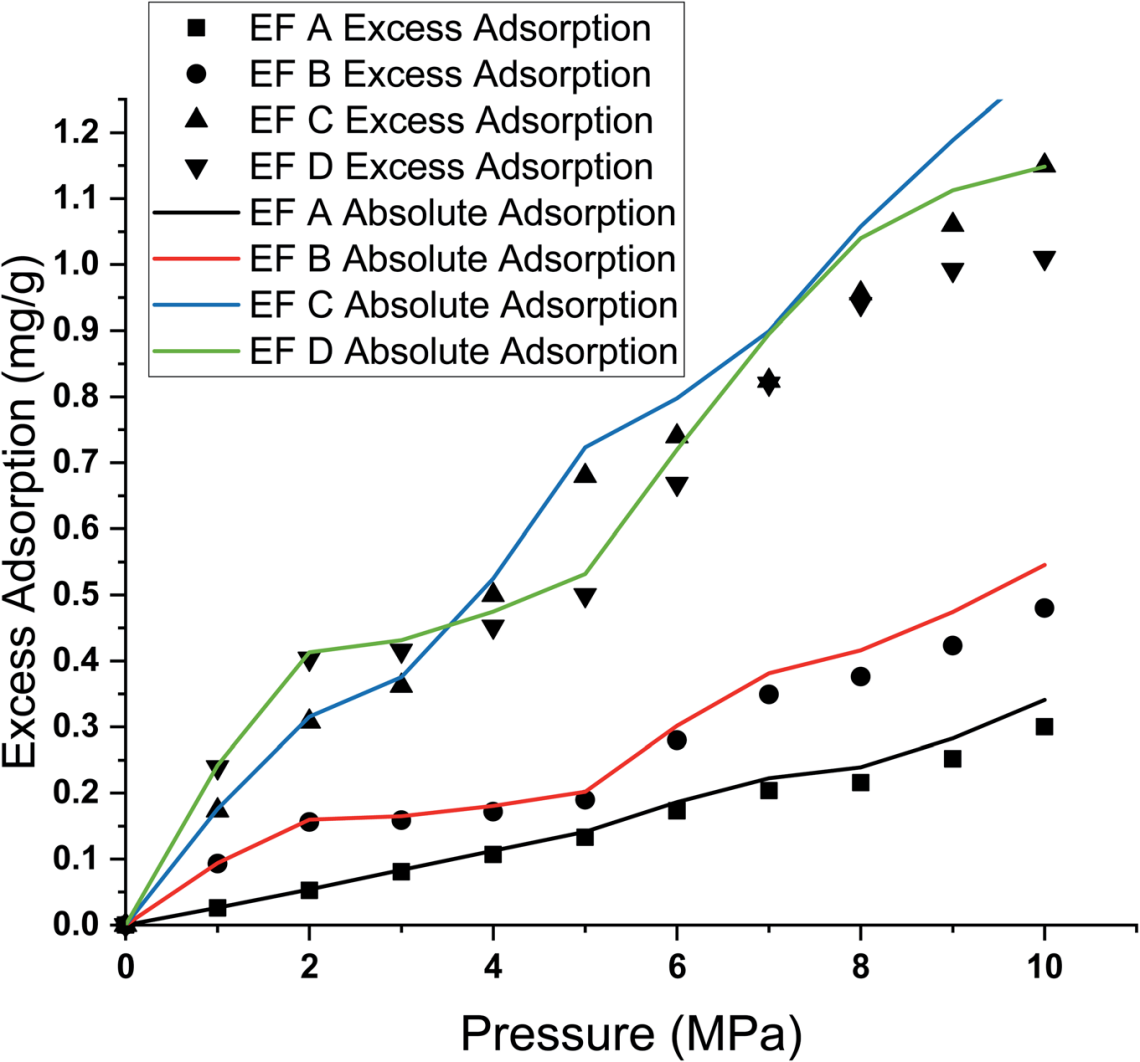
The measured excess and absolute adsorption of shale samples.

The measured adsorption isotherm of supercritical methane on the four shale samples is presented in Fig. 4. The figure shows that the calculated absolute adsorption of supercritical methane is higher than the excess adsorption. This observation indicates that the excess adsorption underestimates the *in situ* adsorption. The value differences between the absolute and excess adsorption enlarge at higher pressure, more apparent after the methane critical pressure of 4.59 MPa. To make a comparison, we chose to look at the percentage of each sample's deviation at 3 MPa (below critical pressure) and 8 MPa (above critical pressure).

The difference in absolute and excess adsorption percentage deviations is much more significant at 8 MPa than at 3 MPa for all samples. The difference in adsorption capacity between excess and absolute adsorption is 3.5% at 3 MPa and 9.6% at 8 MPa (Table 2).

**Table 2:** Comparison of absolute and adsorption capacity of shale samples

Samples	Excess (mg g^−1^)	Absolute (mg g^−1^)	Difference (%)
**Adsorption capacity at 3 MPa**			
EF A	0.081	0.084	3.57
EF B	0.159	0.165	3.64
EF C	0.362	0.375	3.47
EF D	0.416	0.431	3.48

**Adsorption capacity at 8 MPa**			
EF A	0.216	0.239	9.62
EF B	0.376	0.416	9.62
EF C	0.956	1.058	9.64
EF D	0.940	1.040	9.62

The difference between the excess and absolute adsorption capacity has been discussed in detail by the researchers. Theoretically, the adsorption capacity obtained through experimental measurements on porous media is called the Gibbs excess adsorption. In the laboratory, adsorption measurements cannot measure the actual adsorbed quantity. Under supercritical conditions, molecules at an adsorption phase lose translational kinetic energy due to the adsorption potential while maintaining high rotational and vibrational kinetic energy.^80,81^

Therefore, while having a quasi-liquid nature with a fixed molecular spacing, the adsorption phase molecules' gap is slightly larger than liquid molecules at standard critical pressure and temperature. As a result, the density of the supercritical adsorption phase should be greater than the critical density. Consequently, the estimation of adsorption capacity through experimental is not significant to determine the same adsorption capacity of supercritical methane on shale.

The difference between absolute adsorption capacity and excess adsorption was more apparent at high pressure than at lower pressure during isotherm measurement due to the low bulk methane density at relatively low pressure. Thus, any tiny deviations were negligible. However, a higher deviation was observed from absolute adsorption at higher pressure at a constant temperature.^80^

#### Adsorption capacity governing parameters

The adsorption capacity is influenced by the system pressure and the compositions of shale in constant temperature conditions. Specifically, the adsorption of methane increases as the pressure increases. As illustrated in Fig. 4, almost all the shale samples showed positive adsorption capacity correlations with TOC content. For example, sample EF C has the highest adsorption capacity with 1.307 mg g^−1^, followed by EF D with 1.194 mg g^−1^, EF B with 0.546 mg g^−1^, and EF A with 0.455 mg g^−1^. The maximum adsorption capacity value and TOC are tabulated in Table 3.

**Table 3:** TOC and maximum adsorption capacity of Eagle Ford shale samples

Samples	TOC (%)	Maximum adsorption capacity (mg g^−1^)
EF A	10.4	0.341
EF B	9.69	0.546
EF C	14.4	1.307
EF D	10.7	1.148

This correlation implied that the organic content is one of the factors governing methane adsorption on shales. It has been stated that a higher TOC would contribute to a higher adsorption capacity.^16,25,33,34^ The presence of abundant micropores in the organic matter would provide large SSA or sorption sites for the adsorption.

However, in shale, the development of pores is influenced by the diagenesis and variation of shale compositions.^82–85^ Therefore, this may explain the adsorption value found using EF A. According to the literature, there was a positive correlation between the adsorption capacity and TOC readings. However, the discrepancies were observed with EF A being compared with EF B and EF D. EF B has lower TOC and showed higher adsorption capacity with approximately 0.2 mg g^−1^, and EF D with TOC of 10.7% with 0.8 mg g^−1^ higher than EF A adsorption capacity. The observation can be explained as follow.

Several studies on shale samples have shown that shale pore is controlled by the thermal maturity and kerogen type.^86^ Thermal maturity denotes changes in the organic matter through diagenesis when it is subjected to heating. Therefore, vitrinite reflectance (*R*_o_, %) is a critical diagnosing tool for assessing the thermal maturation of organic matter.

Ross and Bustin (2009), in their work with Devonian–Mississippian shale, have found that samples with low TOC (<4.9%) and thermally matured (1.6% < *R*_o_ < 2.5%) have higher adsorption capacity compared to shales with high TOC (>4.9%) and thermally immature (1.2% < *R*_o_).^34^ The same observation was reported in the study, where adsorption capacities on a TOC basis increased with thermal maturity as thermal maturation creates micropores in shale, causing the structural transformation of the shale matrix.

Kerogen type is another important parameter controlling the adsorption capacity in shale. The methane adsorption capacities of kerogen increased according to the type of kerogen. The adsorption capacity is the highest in Type III kerogen, followed by Type II and Type I. The type of kerogen results from thermal maturity. Due to thermal maturation, thermal decarboxylation results in loss of CO_2_ from organic acids, *n*-alkyl fragments break off, and the residual kerogen evolves towards a more condensed structure rich in aromatic and polyaromatic groups.^87,88^ In their work, Zhang *et al.* (2012) stated that aromatic-rich kerogens have a stronger affinity with methane than kerogens with more aliphatic organic matter.^16^

**Fig. 5:**
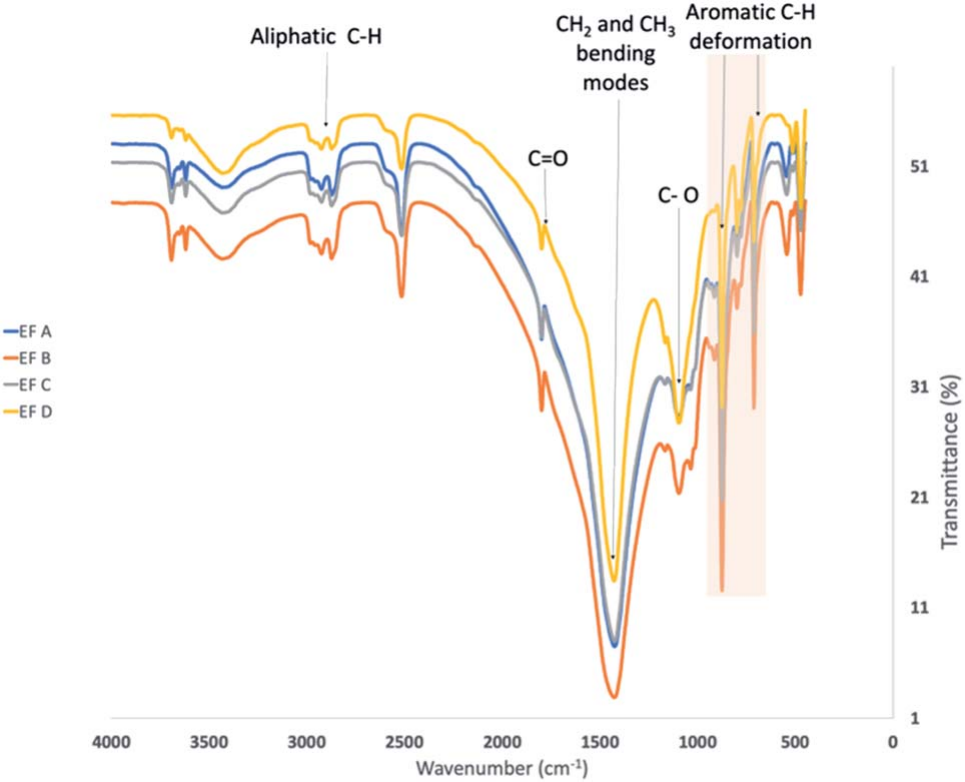
FTIR spectra of the shale samples.

FTIR spectroscopy was used to elucidate the molecular structure of the kerogen by identifying the functional group of kerogens and the types of bonding present. As shown in Fig. 5, several absorption bands of functional groups for shale samples were observed in this study, reflecting the gross structure of kerogen. In addition, absorption bands were identified by comparison with published spectra.^89–91^

In this study, the estimated proportion obtained by visual comparison of the peaks measured from 3000–2800 cm^−1^ is related to the aliphatic C–H stretching region, and from 1700 to 1500 cm^−1^ is attributed to the presence of oxygen-containing bonds C=O for oxygenated groups and aromatic/olefinic region. In addition, peaks were also observed at 1375–1450 cm^−1^ indicating CH_2_ and CH_3_ bending modes and at 700–900 cm^−1^ displaying the aromatic out-of-plane C–H bending signals.

FTIR of spectra of kerogens with increasing maturity exhibit increasing aromatic absorption (700–900 cm^−1^), decreasing in aliphatic absorption (3000–2800 cm^−1^, 1375–1450 cm^−1^) and decreasing in carbonyl and carboxyl absorption (1700 to 1500 cm^−1^). All the samples display low aliphatic C–H stretching band peaks in the 3000–2800 cm^−1^ region. Furthermore, it is noteworthy that responses at regions 700–900 cm^−1^ for aromatic stretching bands demonstrate the presence of organic compounds. EF B samples possess relatively higher aromatic compounds than other samples, which could correlate to the observation of high maximum adsorption capacity compared to EF A samples.

### 4.3 Adsorption isotherms model evaluation

**Fig. 6:**
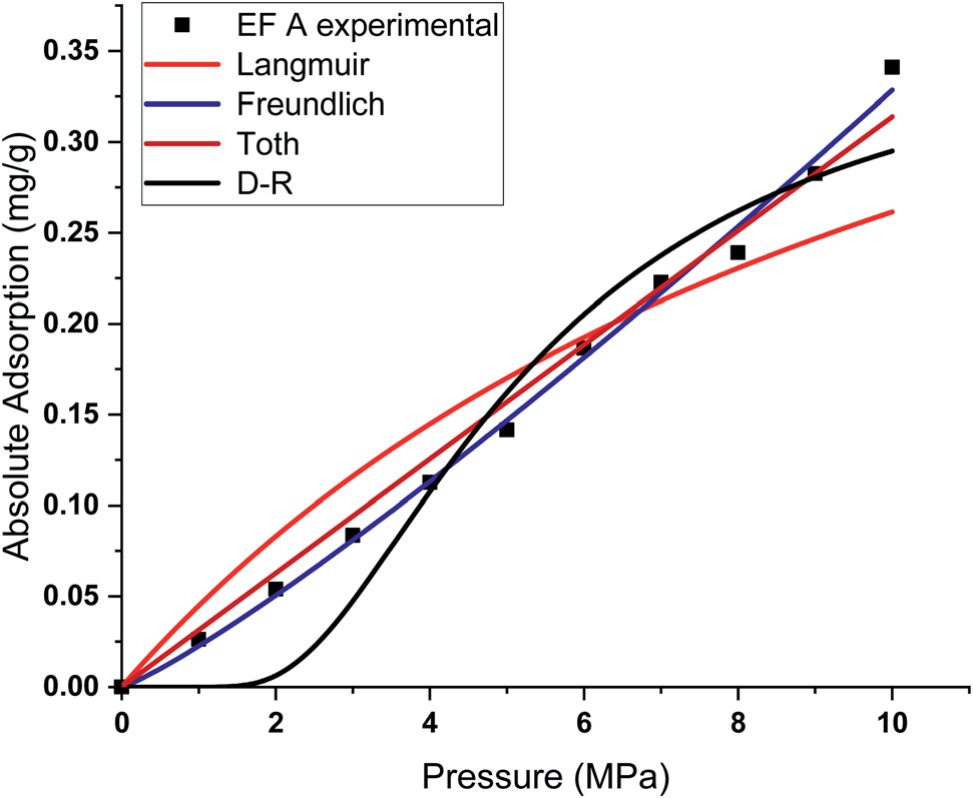
Curve fitting results with the measured absolute adsorption of methane on the Eagle Ford shale samples EF A using Langmuir, Freundlich, Toth, D–R adsorption isotherms.

**Fig. 7:**
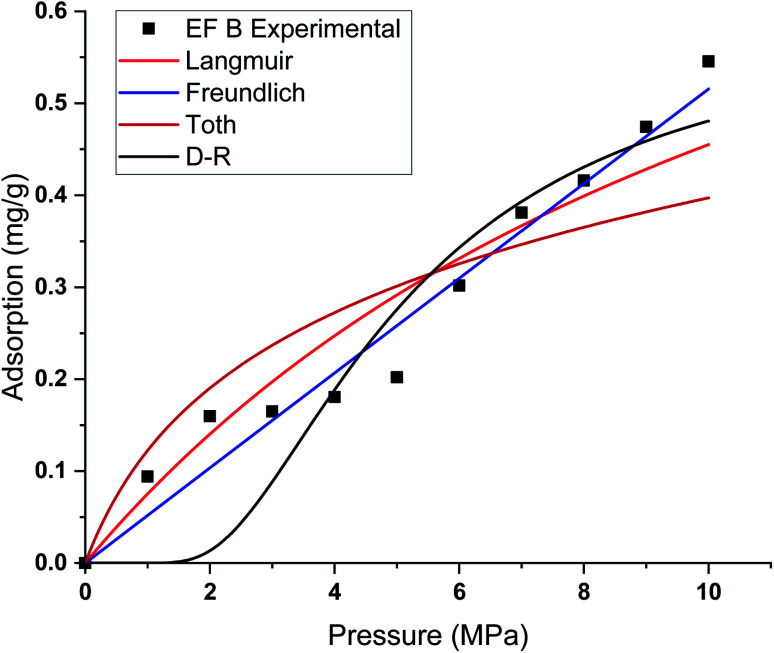
Curve fitting results with the measured absolute adsorption of methane on the Eagle Ford shale samples EF B using Langmuir, Freundlich, Toth, D–R adsorption isotherms.

**Fig. 8:**
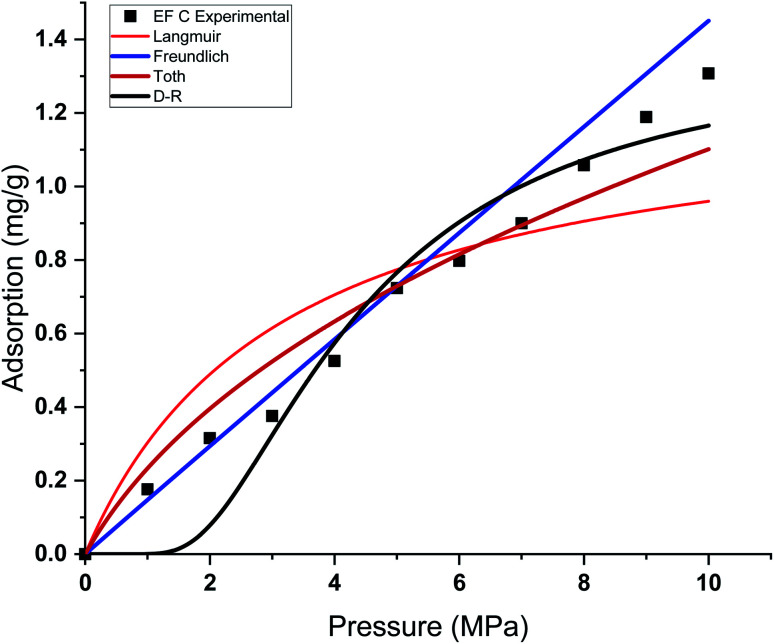
Curve fitting results with the measured absolute adsorption of methane on the Eagle Ford shale samples EF C using Langmuir, Freundlich, Toth, D–R adsorption isotherms.

**Fig. 9:**
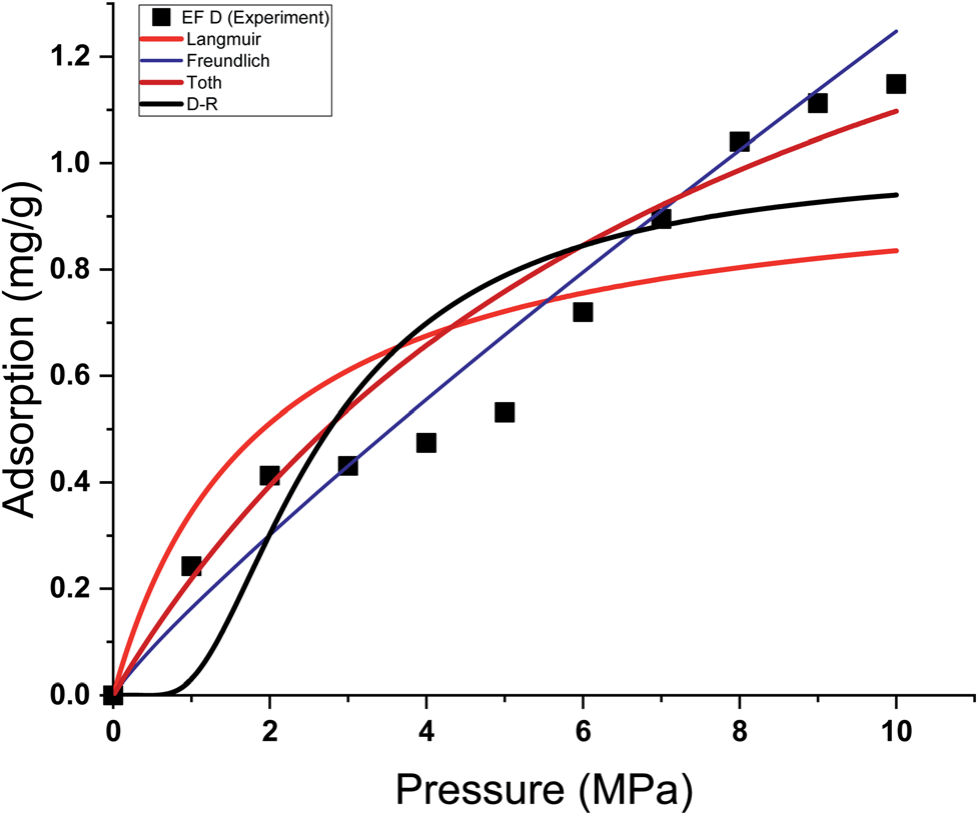
Curve fitting results with the measured absolute adsorption of methane on the Eagle Ford shale samples EF D using Langmuir, Freundlich, Toth, D–R adsorption isotherms.

This study used four different adsorption isotherm models to fit the absolute adsorption capacity obtained experimentally. The fitting parameters and error analysis for each isotherm model with experimental data are shown in Table 4. The fitting of each isotherm model and experimental results are shown in Fig. 6–9, and the error analysis is in Tables 4 and 5.

**Table 4:** Adsorption isotherms fitting parameters

Models	Fitting parameters	Samples name			
		EF A	EF B	EF C	EF D
Langmuir	*q*_m_	0.565	1.036	1.263	0.993
	*K*_L_	0.086	0.078	0.316	0.531
	*R* ^2^	0.89	0.90	0.81	0.70
Freundlich	*K*_F_	0.023	0.052	0.148	0.164
	*n*	0.860	1.002	1.008	1.133
	*R* ^2^	0.99	0.96	0.99	0.94
Toth	*K*_T_	0.443	0.620	0.364	1.533
	*a*_T_	5.810	4.563	1.474	8.061
	*T*	1.036	1.028	2.04	1.293
	*R* ^2^	0.99	0.99	0.99	0.99
D–R	*q*_s_	0.370	0.595	1.365	1.005
	*k*_ads_	7.586	7.104	5.320	2.231
	*R* ^2^	0.92	0.84	0.92	0.76

**Table 5:** Adsorption isotherms non-linear regression error analysis

Models	Fitting parameters	Samples name			
		EF A	EF B	EF C	EF D
Langmuir	*χ* ^2^	0.001	0.004	0.015	0.042
	RMSE	0.033	0.054	0.098	0.168
	ARE (%)	1.90	13.3	10.7	8.8
Freundlich	*χ* ^2^	0.001	0.001	0.008	0.007
	RMSE	0.007	0.031	0.082	0.077
	ARE (%)	2.2	2.8	8.70	4.02
Toth	*χ* ^2^	0.001	0.004	0.017	0.015
	RMSE	0.012	0.056	0.074	0.104
	ARE (%)	2.2	0.6	2.5	2.2
D–R	*χ* ^2^	0.001	0.005	0.016	0.033
	RMSE	0.027	0.005	0.113	0.165
	ARE (%)	4.4	8.5	4.0	1.4

Each sample has a different effect on the performance of each isotherm model. The *χ*^2^, RMSE, and ARE error analyses were conducted to analyse the feasibility of adsorption models with experimental adsorption measurements. The ARE is utilised as a major indication to assess the accuracy of the adsorption model with experimental measurements of each sample.

In general, the *χ*^2^ for all the samples was relatively lower, with less than 0.05 for all the samples. The Freundlich model has the lowest *χ*^2^ values, followed by Toth and D–R models. In contrast, the Langmuir model exhibits the highest *χ*^2^ value, particularly for EF D samples. The RMSE values of the adsorption data demonstrate the relationship between experimental and model data, with low RMSE obtained from the Freundlich, Toth, and D–R models, supporting the best-fit isotherm models for non-linear regression analysis.

The ARE analysis showed that the Toth model has the lowest values compared to other models, 0.6% for EF B, 2.5% for EF A, and 2.2% for EF A and EF D, respectively. The Freundlich model rank second, followed by the D–R model. Meanwhile, the Langmuir model shows significant error for the fitting model with ARE values between 10 and 13%. The order of error of the isotherm model of each sample is tabulated in Table 6.

**Table 6:** Order of error of isotherm model with experimental data

Samples	Order of error of isotherm model
EF A	Langmuir > Freundlich = Toth > D–R
EF B	Toth > Freundlich > D–R > Langmuir
EF C	Toth > D–R > Freundlich > Langmuir
EF D	D–R > Toth > Freundlich > Langmuir

The theoretical assumptions could explain the deviations of adsorption data from the Langmuir and that the adsorbent surface must be homogenous, which did not fulfil in the case of heterogeneous shale characteristics. Shale samples owned broad pore size distributions with pore sizes ranging between 2 nm to 50 nm. The influence of pore walls is significant, leading to the high potential energy between methane molecules and shale surface, the effect more significant in smaller pores. The violation of the homogeneity assumption with the Langmuir model could have been responsible for inaccurate fittings to methane adsorption data on shales. This study also shows that all the models show great prediction with absolute adsorption capacity measurements at low pressure compared to high pressure.

This study also shows that the methane adsorption equilibrium at high pressure and temperature in shale did not obey the Type I adsorption isotherm (*e.g.* Langmuir and Freundlich). This resulted from the fact that methane adsorption did not follow the Type I isotherm. This isotherm is defined as the adsorption of gas molecules to adsorbents limited to one molecular layer.^92^ Meanwhile, the Freundlich model was developed from the theory of heterogeneous adsorbent on the surface of adsorbate, thus showing a great ability to depict the adsorption in shale. However, discrepancies from the model were observed in the samples with higher TOC values. Samples with higher TOC own a larger volume of pores compared to low TOC value samples. The presence of these pores could change the adsorption behaviour in shale. Therefore, methane adsorption in shale could be categorised as Type II (multilayer, Toth) or Type III (pore filling, D–R). This explained the observation from experiment measurement that shows higher agreement with D–R or Toth adsorption isotherm model.

#### Comparing the adsorption isotherms with TOC

**Fig. 10:**
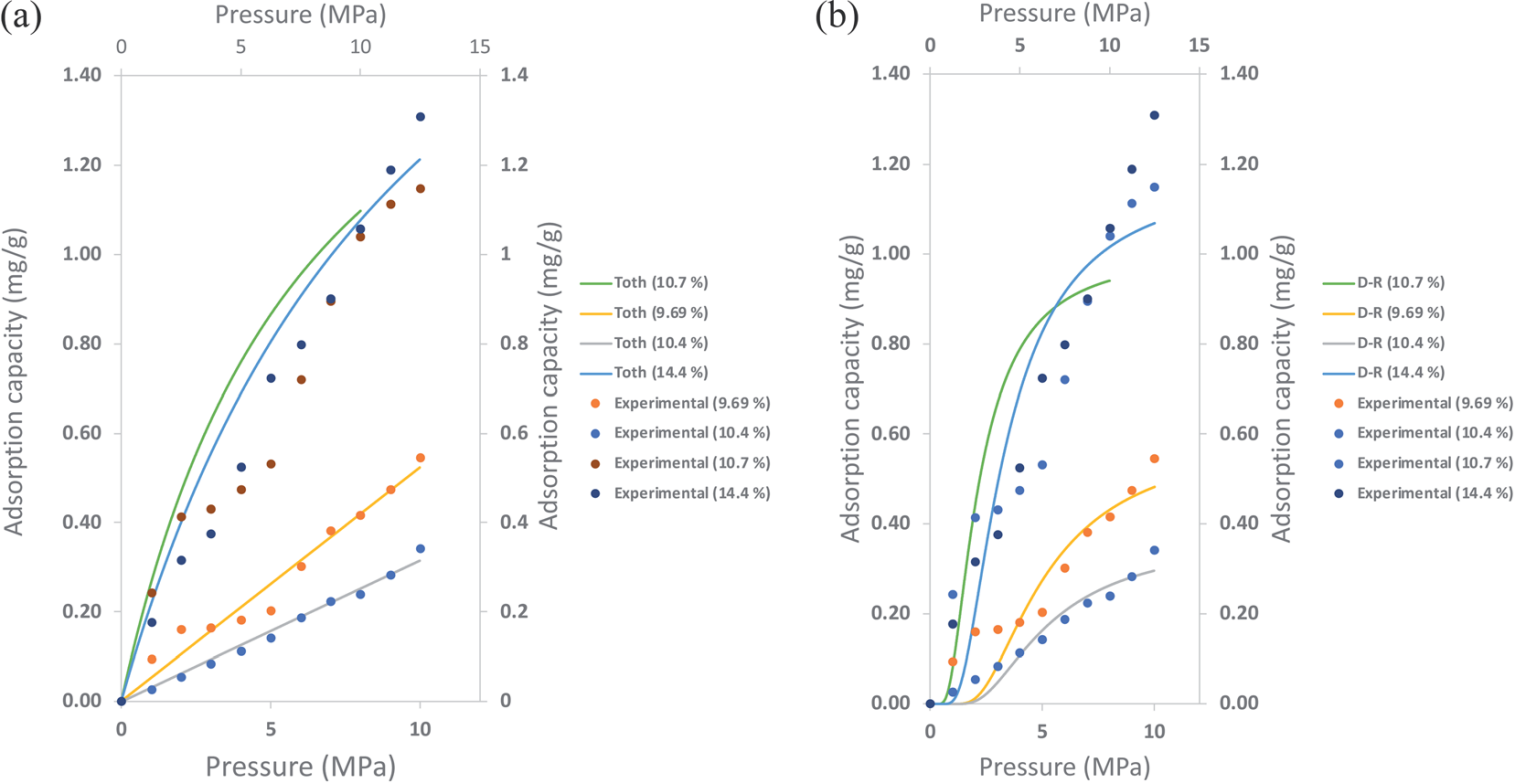
Correlation of the adsorption capacity from experimental measurement, adsorption isotherm with samples TOC; (a) Toth isotherm (b) D–R isotherm.

The adsorption capacities of shale samples were calculated using the D–R and Toth adsorption isotherms to determine the correlations with the TOC of the samples. The correlations of calculated adsorption capacity with TOC are graphically described in Fig. 10.

In Fig. 10, both isotherms perfectly depict the adsorption isotherm for samples with 9.69% and 10.4% TOC values. However, Toth and D–R adsorption isotherms underestimate the adsorption capacity for samples with higher TOC, 10.7% and 14.4%. The discrepancies were more apparent for TOC of 14.4%, 7% for calculation with Toth isotherm, and 18% with D–R isotherm. The percentage of differences in the maximum adsorption capacities obtained through both methods are tabulated in Table 7.

**Table 7:** Comparison of the maximum adsorption capacity obtained through experimental measurements and TOC values

TOC (%)	Maximum adsorption capacity (mg g^−1^)				
	Experimental measurements	Toth	Differences (%)	D–R	Differences (%)
9.69	0.546	0.524	4	0.512	6
10.4	0.341	0.334	2	0.321	6
10.7	1.148	1.097	4	0.94	18
14.4	1.307	1.213	7	1.068	18

These isotherms' ability to determine methane adsorption on shale has been discussed in the literature previously. However, the limitations of these isotherms depicting the adsorption towards the TOC of shale are rarely discussed. As the TOC increases, the ability of these isotherms to predict the adsorption shows discrepancies, especially at high pressure. Taking into account that these measurements were also conducted at 120 °C. It shows that current empirical adsorption can predict methane adsorption in shale to a certain degree. Thus, an in-depth study is required to address the heterogeneous properties of shale.

## 5 Conclusions

This study conducted the methane adsorption isotherms of four shale samples from Eagle Ford shale reservoirs. The adsorption isotherm measurements were performed at 120 °C and up to 10 MPa. The shale samples were characterised, and the adsorption models were used to describe the methane adsorption behaviour. According to the results obtained, the main conclusion from this work is summarised as follows;

(1) The TOC of the shale sample values ranged from 9.69% to 14.4%. The results show that the SSA positively correlates with TOC creating favourable gas adsorption and storage conditions.

(2) The calculated absolute adsorption of supercritical methane is higher than the excess adsorption. Therefore, the difference between the absolute and excess adsorption percentage of deviations is more significant at a pressure higher than the critical methane pressure. For example, the difference between excess and absolute adsorption is 3.5% at a lower pressure than 9.6% at higher pressure.

(3) The adsorption isotherm of methane increased with pressure, and almost all the shale samples showed positive adsorption capacity correlations with TOC content. Sample EF 4 has the highest adsorption capacity with 1.307 mg g^−1^, followed by EF 5 with 1.148 mg g^−1^, EF 2 with 0.546 mg g^−1^, and EF 1 with 0.341 mg g^−1^. The discrepancies in the value obtained could result from the pore properties caused by thermal maturity and kerogen type.

(4) FTIR analysis shows EF B samples possess relatively higher aromatic compounds than other samples, which could correlate to the observation of high maximum adsorption capacity compared to EF A samples.

(5) The order of adsorption isotherm fitting with experimental data is Toth > D–R = Freundlich > Langmuir. Error analysis shows Toth has the lowest values compared to other models, 0.6% for EF B, 2.5% for EF A, and 2.2% for EF A and EF D, respectively. The Langmuir model shows significant error for the fitting model with ARE values between 10 and 13%.

(6) However, discrepancies from the Freundlich model were observed in the samples with higher TOC values. Samples with higher TOC own a larger volume of pores compared to low TOC value samples.

(7) Methane adsorption in shale could be categorised as Type II (multilayer, Toth) or Type III (pore filling, D–R).

(8) The D–R and Toth isotherms perfectly depict the adsorption isotherm for samples with 9.69% and 10.4% TOC values. However, Toth and D–R adsorption isotherms underestimate the adsorption capacity for samples with higher TOC (10.7% and 14.4%). The discrepancies were more apparent for TOC of 14.4%, 7% for calculation with Toth isotherm, and 18% with D–R isotherm.

The ability of these isotherms to determine the adsorption of methane on shale has been studied by researchers. However, the limitations of these isotherms depicting the adsorption towards the TOC of shale are rarely discussed. Nevertheless, the data has shown certain agreement in predicting the adsorption capacity through this study. Thus, in-depth research is required to address the properties of shale.

## Author contributions

Aminah Qayyimah, Dzeti, Belladonna, Khaled: ideas, formulation and conceptualisation of the study. Aminah Qayyimah, Dzeti, Belladonna: design of methodology and development of data measurement. Aminah Qayyimah, Dzeti: verification and validation of experimental and other research output. Aminah Qayyimah: preparation, creation and presentation of the data-corresponding author. Dzeti, Belladonna, Khaled: critical review, commentary or revision – including pre-or post-publication stages.

## Conflicts of interest

There are no conflicts of interest to declare.

## Supplementary Material

## References

[cit1] Ahmad T., Zhang D. (2020). A critical review of comparative global historical energy consumption and future demand: the story told so far. Energy Rep..

[cit2] BP Global , BP Statistical Review of World Energy 2020, 2020, available: https://bp.com/content/dam/bp/business-sites/en/global/corporate/pdfs/energy-economics/statistical-review/bp-stats-review-2020-full-report.pdf

[cit3] U. S. E. I. A. (EIA) , International Energy Outlook (IEO) 2019, U. S. Energy Information Administration, 2019

[cit4] Kim J. H., Lee Y. G. (2020). Progress of Technological Innovation of the United States’ Shale Petroleum Industry Based on Patent Data Association Rules. Sustainability.

[cit5] Wang Q., Chen X., Jha A. N., Rogers H. (2014). Natural gas from shale formation–the evolution, evidences and challenges of shale gas revolution in United States. Renewable Sustainable Energy Rev..

[cit6] Jiang Z., Duan H., Liang C., Wu J., Zhang W., Zhang J. (2017). Classification of hydrocarbon-bearing fine-grained sedimentary rocks. J. Earth Sci..

[cit7] Curtis J. B. (2002). Fractured shale-gas systems. Am. Assoc. Pet. Geol. Bull..

[cit8] Montgomery S. L., Jarvie D. M., Bowker K. A., Pollastro R. M. (2005). Mississippian Barnett Shale, Fort Worth basin, north-central Texas: gas-shale play with multi–trillion cubic foot potential. Am. Assoc. Pet. Geol. Bull..

[cit9] Zhang T. (2019). *et al.*, The transport behaviors of oil in nanopores and nanoporous media of shale. Fuel.

[cit10] Alfi M. , *et al.*, How to improve our understanding of gas and oil production mechanisms in liquid-rich shale, 2014

[cit11] Sinha S. , et al., Advances in measurement standards and flow properties measurements for tight rocks such as shales, in SPE/EAGE European Unconventional Resources Conference & Exhibition-From Potential to Production, 2012, p. cp-285

[cit12] Kuila U., Prasad M. (2013). Specific surface area and pore-size distribution in clays and shales. Geophys. Prospect..

[cit13] Gensterblum Y. (2015). et al., Gas transport and storage capacity in shale gas reservoirs - a review. Part A: transport processes. Journal of Unconventional Oil and Gas Resources.

[cit14] Zou C. (2010). et al., Geological characteristics and resource potential of shale gas in China. Pet. Explor. Dev..

[cit15] Javadpour F., Fisher D., Unsworth M. (2007). Nanoscale gas flow in shale gas sediments. J. Can. Pet. Technol..

[cit16] Zhang T., Ellis G. S., Ruppel S. C., Milliken K., Yang R. (2012). Effect of organic-matter type and thermal maturity on methane adsorption in shale-gas systems. Org. Geochem..

[cit17] Kang S. M., Fathi E., Ambrose R. J., Akkutlu I. Y., Sigal R. F. (2011). Carbon dioxide storage capacity of organic-rich shales. SPE J..

[cit18] Gregg S. J., Sing K. S. W., Salzberg H. W. (1967). Adsorption Surface Area and Porosity. J. Electrochem. Soc..

[cit19] Chalmers G. R. L., Bustin R. M. (2008). Lower Cretaceous gas shales in northeastern British Columbia, part I: geological controls on methane sorption capacity. Bull. Can. Pet. Geol..

[cit20] Gasparik M., Bertier P., Gensterblum Y., Ghanizadeh A., Krooss B. M., Littke R. (2014). Geological controls on the methane storage capacity in organic-rich shales. Int. J. Coal Geol..

[cit21] Chen L. (2019). et al., Mechanisms of shale gas adsorption: evidence from thermodynamics and kinetics study of methane adsorption on shale. Chem. Eng. J..

[cit22] Tang X., Ripepi N., Luxbacher K., Pitcher E. (2017). Adsorption Models for Methane in Shales: Review, Comparison, and Application. Energy Fuels.

[cit23] Dang W. (2020). et al., Isotherms, thermodynamics and kinetics of methane-shale adsorption pair under supercritical condition: implications for understanding the nature of shale gas adsorption process. Chem. Eng. J..

[cit24] Qajar A., Daigle H., Prodanović M. (2015). Methane dual-site adsorption in organic-rich shale-gas and coalbed systems. Int. J. Coal Geol..

[cit25] Lu X.-C., Li F.-C., Watson A. T. (1995). Adsorption measurements in Devonian shales. Fuel.

[cit26] Li J. (2015). et al., Key factors controlling the gas adsorption capacity of shale: a study based on parallel experiments. Appl. Geochem..

[cit27] Zhou S., Xue H., Ning Y., Guo W., Zhang Q. (2018). Experimental study of supercritical methane adsorption in Longmaxi shale: insights into the density of adsorbed methane. Fuel.

[cit28] la Fuente J. D., Santiago J., Román A., Dumitrache C., Casasanto D. (2014). Matrix Permeability Measurements of Gas Shales: Gas Slippage and Adsorption as Sources of Systematic Error. Psychological Science.

[cit29] Rani S., Prusty B. K., Padmanabhan E., Pal S. K. (2019). Applicability of various adsorption isotherm models on adsorption of methane and CO_2_ on Indian shales. Environ. Prog. Sustainable Energy.

[cit30] Rexer T. F., Mathia E. J., Aplin A. C., Thomas K. M. (2020). Supercritical methane adsorption and storage in pores in shales and isolated kerogens. SN Appl. Sci..

[cit31] Ma Y., Jamili A. (2016). Modeling the density profiles and adsorption of pure and mixture hydrocarbons in shales. Journal of Unconventional Oil and Gas Resources.

[cit32] Pan L., Xiao X., Zhou Q. (2016). The influence of soluble organic matter on shale reservoir characterisation. Journal of Natural Gas Geoscience.

[cit33] Clarkson C. R., Haghshenas B. (2013). Modeling of supercritical fluid adsorption on organic-rich shales and coal. Society of Petroleum Engineers – SPE USA Unconventional Resources Conference.

[cit34] Ross D. J. K., Bustin R. M. (2009). The importance of shale composition and pore structure upon gas storage potential of shale gas reservoirs. Mar. Pet. Geol..

[cit35] Chen L., Jiang Z., Liu K., Gao F. (2017). Quantitative characterisation of micropore structure for organic-rich lower silurian shale in the upper yangtze platform, South China: implications for shale gas adsorption capacity. Advances in Geo-Energy Research.

[cit36] Li Z., Jin Z., Firoozabadi A. (2014). Phase Behavior and Adsorption of Pure Substances and Mixtures and Characterisation in Nanopore Structures by Density Functional Theory. SPE J..

[cit37] Zhou J., Jin Z., Luo K. H. (2019). Effects of Moisture Contents on Shale Gas Recovery and CO_2_ Sequestration. Langmuir.

[cit38] Guo F., Wang S., Feng Q., Yao X., Xue Q., Li X. (2020). Adsorption and absorption of supercritical methane within shale kerogen slit. J. Mol. Liq..

[cit39] Tang X., Ripepi N., Stadie N. P., Yu L., Hall M. R. (2016). A dual-site Langmuir equation for accurate estimation of high pressure deep shale gas
resources. Fuel.

[cit40] Rani S., Padmanabhan E., Prusty B. K. (2019). Review of gas adsorption in shales for enhanced methane recovery and CO_2_ storage. J. Pet. Sci. Eng..

[cit41] Barsotti E., Lowry E., Piri M., Chen J. H. (2020). Using Capillary Condensation and Evaporation Isotherms to Investigate Confined Fluid Phase Behavior in Shales. E3S Web Conf..

[cit42] Yu W., Sepehrnoori K., Patzek T. W. (2016). Modeling gas adsorption in Marcellus shale with Langmuir and bet isotherms. SPE J..

[cit43] Li C., Li L., Kang T. (2019). Measurement and modeling of the adsorption isotherms of CH_4_ and C_2_H_6_ on shale samples. RSC Adv..

[cit44] Mohd Aji A. Q. , Maulianda B., Mohshim D. F., Elraeis K. A. and Ku Ishak K. E. H., Supercritical Methane Adsorption in Shale: Isothermal Adsorption and Desorption of Eagle Ford Shale Gas, 2022 10.1039/d2ra03367d PMC9284539 35919182

[cit45] Li D., Zhang L., Wang J. Y., Lu D. (2016). Composition-Transient Analysis in Shale-Gas Reservoirs with Consideration of Multicomponent Adsorption. SPE J..

[cit46] Meng M., Zhong R., Wei Z. (2020). Prediction of methane adsorption in shale: classical models and machine learning based models. Fuel.

[cit47] Schamel S. , Shale gas resources of Utah: assessment of previously undeveloped gas discoveries, Utah Geological Survey, 2006

[cit48] Wang Z., Li Y., Guo P., Meng W. (2016). Analysing the Adaption of Different Adsorption Models for Describing the Shale Gas Adsorption Law. Chem. Eng. Technol..

[cit49] Tang X., Ripepi N., Stadie N. P., Yu L., Hall M. R. (2016). A dual-site Langmuir equation for accurate estimation of high pressure deep shale gas resources. Fuel.

[cit50] Varma A. K., Khatun M., Mendhe V. A., Hazra B., Singh B. D., Dayal A. M. (2015). Petrographic characterisation and Langmuir volume of shales from Raniganj coal basin, India. J. Geol. Soc. India.

[cit51] Li J., Ma Y., Huang K., Lu S., Yin J., Zhang Y. (2017). Comprehensive polynomial simulation and prediction for Langmuir volume and Langmuir pressure of shale gas adsorption using multiple factors. Mar. Pet. Geol..

[cit52] Tang X., Ripepi N., Stadie N. P., Yu L., Hall M. R. (2016). A dual-site Langmuir equation for accurate estimation of high pressure deep shale gas resources. Fuel.

[cit53] Alafnan S., Awotunde A., Glatz G., Adjei S., Alrumaih I., Gowida A. (2021). Langmuir adsorption isotherm in unconventional resources: applicability and limitations. J. Pet. Sci. Eng..

[cit54] Zhang F., Emami-Meybodi H. (2021). Analysis of early-time production data from multi-fractured shale gas wells by considering multiple transport mechanisms through nanopores. J. Pet. Sci. Eng..

[cit55] Ekundayo J. M., Rezaee R. (2019). Numerical Simulation of Gas Production from Gas Shale Reservoirs—Influence of Gas Sorption Hysteresis. Energies.

[cit56] Boyer C., Kieschnick J., Suarez-Rivera R., Lewis R. E., Waters G. (2006). Producing gas from its source. Oilfield Rev..

[cit57] Berawala D. S., Andersen P. Ø. (2020). Numerical investigation of Non-Darcy flow regime transitions in shale gas production. J. Pet. Sci. Eng..

[cit58] Alexander T. (2011). *et al.*, Shale gas revolution. Oilfield Rev..

[cit59] Rani S., Prusty B. K., Pal S. K. (2015). Methane adsorption and pore characterisation of Indian shale samples. Journal of Unconventional Oil and Gas Resources.

[cit60] Ross D. J. K., Bustin R. M. (2007). Impact of mass balance calculations on adsorption capacities in microporous shale gas reservoirs. Fuel.

[cit61] Freundlich H. M. F. (1906). Over the adsorption in solution. J. Phys. Chem..

[cit62] Li A. (2017). *et al.*, Investigation of the methane adsorption characteristics of marine shale: a case study of Lower Cambrian Qiongzhusi Shale in eastern Yunnan Province, South China. Energy Fuels.

[cit63] Zhu H., Jia A., Wei Y., Jia C., Jin Y., Yuan H. (2018). Characteristics of microscopic pore structure and methane adsorption capacity of shale in the Longmaxi Formation in the Zhaotong area. Pet. Geol. Recovery Effic..

[cit64] Toth J. (1971). State equation of the solid-gas interface layers. Acta Chim. Hung..

[cit65] Dubinin M. M., Astakhov V. A. (1971). Development of the concepts of volume filling of micropores in the adsorption of gases and vapors by microporous adsorbents. Bull. Acad. Sci. USSR, Div. Chem. Sci..

[cit66] Hu Q., Zhang Z. (2019). Application of Dubinin–Radushkevich isotherm model at the solid/solution interface: a theoretical analysis. J. Mol. Liq..

[cit67] Rexer T. F. T., Benham M. J., Aplin A. C., Thomas K. M. (2013). Methane adsorption on shale under simulated geological temperature and pressure conditions. Energy Fuels.

[cit68] Song X., Lü X., Shen Y., Guo S., Guan Y. (2018). A modified supercritical Dubinin–Radushkevich model for the accurate estimation of high pressure methane adsorption on shales. Int. J. Coal Geol..

[cit69] Chen L. (2017). et al., Application of Langmuir and Dubinin–Radushkevich models to estimate methane sorption capacity on two shale samples from the Upper Triassic Chang 7 Member in the southeastern Ordos Basin, China. Energy Explor. Exploit..

[cit70] Foo K. Y., Hameed B. H. (2010). Insights into the modeling of adsorption isotherm systems. Chem. Eng. J..

[cit71] Wang X. , Chapter 1 - Introduction, in Lacustrine Shale Gas, ed. X. Wang, Gulf Professional Publishing, 2017, pp. 1–40, 10.1016/B978-0-12-813300-2.00001-5

[cit72] Tan J. (2014). et al., Shale gas potential of the major marine shale formations in the Upper Yangtze Platform, South China, Part II: methane sorption capacity. Fuel.

[cit73] Saif T., Lin Q., Bijeljic B., Blunt M. J. (2017). Microstructural imaging and characterisation of oil shale before and after pyrolysis. Fuel.

[cit74] Thommes M. (2010). Physical adsorption characterisation of nanoporous materials. Chem.-Ing.-Tech..

[cit75] Murugesu M. P. (2017). Pore structure analysis using subcritical gas adsorption method. SPE Annu. Tech. Conf. Exhib..

[cit76] Ayawei N., Ebelegi A. N., Wankasi D. (2017). Modelling and Interpretation of Adsorption Isotherms. J. Chem..

[cit77] Sing K. S. W. , Characterization of Porous Solids: An Introductory Survey, in Characterisation of Porous Solids II, ed. F. Rodriguez-Reinoso, J. Rouquerol, K. S. W. Sing and K. K. Unger, Elsevier, 1991, vol. 62, pp. 1–9, 10.1016/S0167-2991(08)61303-8

[cit78] Ojha S. P., Misra S., Tinni A., Sondergeld C., Rai C. (2017). Pore connectivity and pore size distribution estimates for Wolfcamp and Eagle Ford shale samples from oil, gas and condensate windows using adsorption-desorption measurements. J. Pet. Sci. Eng..

[cit79] Altawati F., Emadi H., Khalil R. (2021). An experimental study to investigate the physical and dynamic elastic properties of Eagle Ford shale rock samples. J. Pet. Explor. Prod..

[cit80] Xue P., Zhang L., Liang Q., Sun X., Zhao Q., Qi P. (2020). Thermodynamic characteristics of CH_4_ adsorption by continental shale: a case study of the Upper Triassic Yanchang shale in the Yanchang Gasfield, Ordos Basin. Nat. Gas Ind..

[cit81] Myers A. L., Monson P. A. (2014). Physical adsorption of gases: the case for absolute adsorption as the basis for thermodynamic analysis. Adsorption.

[cit82] Taotao C., Deng M., Cao Q., Yanran H., Yu Y., Cao X. (2021). Pore formation and evolution of organic-rich shale during the entire hydrocarbon generation process: examination of artificially and naturally matured samples. J. Nat. Gas Sci. Eng..

[cit83] Liu W., Liu J., Cai M., Luo C., Shi X., Zhang J. (2017). Pore evolution characteristic of shale in the Longmaxi Formation, Sichuan Basin. Pet. Res..

[cit84] Cui J. W., Zhu R. K., Cui J. G. (2013). Relationship of porous evolution and residual hydrocarbon: evidence from modeling experiment with geological constrains. Acta Geol. Sin..

[cit85] Cao T., Deng M., Cao Q., Huang Y., Yu Y., Cao X. (2021). Pore formation and evolution of organic-rich shale during the entire hydrocarbon generation process: examination of artificially and naturally matured samples. J. Nat. Gas Sci. Eng..

[cit86] Li X. (2016). et al., An insight into the mechanism and evolution of shale reservoir characteristics with over-high maturity. Journal of Natural Gas Geoscience.

[cit87] Rezaee R. , Fundamentals of gas shale reservoirs, John Wiley & Sons, 2015

[cit88] Petsch S. , Kerogen, in Encyclopedia of Geochemistry: A Comprehensive Reference Source on the Chemistry of the Earth, ed. W. M. White, Springer International Publishing, Cham, 2018, pp. 771–775, 10.1007/978-3-319-39312-4_163

[cit89] Tanykova N., Petrova Y., Kostina J., Kozlova E., Leushina E., Spasennykh M. (2021). Study of organic matter of unconventional reservoirs by IR spectroscopy and IR microscopy. Geosciences.

[cit90] Wang S.-H., Griffiths P. R. (1985). Resolution enhancement of diffuse reflectance IR spectra of coals by Fourier self-deconvolution: 1. CH stretching and bending modes. Fuel.

[cit91] Chen Y., Mastalerz M., Schimmelmann A. (2014). Heterogeneity of shale documented by micro-FTIR and image analysis. J. Microsc..

[cit92] Lowell S. and Shields J. E., Adsorption isotherms, in Powder Surface Area and Porosity, Springer Netherlands, Dordrecht, 1984, pp. 11–13, 10.1007/978-94-009-5562-2_3

